# Turnover rate of cerebrospinal fluid in female sheep: changes related to different light-dark cycles

**DOI:** 10.1186/1743-8454-6-9

**Published:** 2009-08-04

**Authors:** Jean-Claude Thiéry, Didier Lomet, Sylvain Bougoin, Benoit Malpaux

**Affiliations:** 1 UMR: INRA – CNRS – Université François Rabelais de Tours – Haras nationaux 37380 Nouzilly, France

## Abstract

**Background:**

Sheep are seasonal breeders. The key factor governing seasonal changes in the reproductive activity of the ewe is increased negative feedback of estradiol at the level of the hypothalamus under long-day conditions. It has previously been demonstrated that when gonadotropin secretions are inhibited during long days, there is a higher concentration of estradiol in the cerebrospinal fluid (CSF) than during short days. This suggests an involvement of the CSF and choroid plexus in the neuroendocrine regulatory loop, but the mechanisms underlying this phenomenon remain unknown. One possible explanation of this difference in hormonal content is an effect of concentration or dilution caused by variations in CSF secretion rate. The aim of this study was thus to investigate changes in the CSF turnover rate related to light-dark cycles.

**Methods:**

The turnover rate of the CSF was estimated by measuring the time taken for the recovery of intraventricular pressure (IVP) after removal of a moderate volume (0.5 to 2 ml) of CSF (slope in mmHg/min). The turnover rate was estimated three times in the same group of sheep: during a natural period of decreasing day-length corresponding to the initial period when gonadotropin activity is stimulated (SG1), during a long-day inhibitory period (IG), and finally during a short-day stimulatory period (SG2).

**Results:**

The time taken and the speed of recovery of initial IVP differed between groups: 8 min 30 sec, 0.63 ± 0.07 mmHg/min(SG1), 11 min 1 sec, 0.38 ± 0.06 mmHg/min (IG) and 9 min 0 sec, 0.72 ± 0.15 mmHg/min (SG2). Time changes of IVP differed between groups (ANOVA, p < 0.005, SG1 different from IG, *p *< 0.05). The turnover rate in SG2: 183.16 ± 23.82 μl/min was not significantly different from SG1: 169. 23 ± 51.58 μl/min (Mann-Whitney test, *p *= 0.41), but was significantly different from IG: 71.33 ± 16.59 μl/min (*p *= 0.016).

**Conclusion:**

This study shows that the turnover rate of CSF in ewes changes according to the light-dark cycle; it is increased during short day periods and reduced in long day periods. This phenomenon could account for differences in hormonal concentrations in the CSF in this seasonal species.

## Background

The choroid plexus has two main functions: it provides a barrier between the blood and the cerebrospinal fluid (CSF), and it is the major secretory source of CSF [1,2]. CSF allows the brain to "float" within the cranium and helps drain metabolites from the brain by its "sink" action. Once in the CSF, metabolites and xenobiotics can be transported to the blood via the choroid plexus [3-5]. Conversely, due to active transport from the blood and intense mixing in the ventricles [6], the CSF provides possible access to the brain for several molecules such as certain hormones, including transthyretin [7], leptin [8] and melatonin [9] (see also [10-12] for review). It is therefore, tempting to speculate that physiological changes in the choroid plexus can cause variations in the composition of the CSF which could modulate the central action of peripheral factors.

Sheep in temperate latitudes are seasonal breeders. Among the different seasonal cues, photoperiod is the most reliable parameter and is used by animals as an indication of the season. The photoperiodic information is transduced into neuroendocrine changes through variations in melatonin secretion from the pineal gland. As a result of these variations in ewes, the female sheep, the key phenomenon governing seasonal changes in gonadotropin is an increase in the negative feedback exerted by estradiol within the hypothalamus during long days [13]. We previously found a higher concentration of progesterone and estradiol in the CSF of ovariectomized, steroid-treated ewes during long than during short days, when gonadotropin secretion is inhibited or stimulated, respectively [14,15]. This photoperiodically-induced increase in steroids in the CSF, which requires the presence of the pineal gland [15], could be related to a more general mechanism, similar to the photoperiodically regulated passage from blood to CSF of leptin, a protein hormone, found in male sheep [16]. This recently-described phenomenon illustrates the possible involvement of the CSF and choroid plexus in the neuroendocrine regulatory loop. However, the mechanisms underlying these changes in hormonal concentration in the CSF remain unknown. Notably, the concentration of a molecule in one compartment reflects its access and clearance, but could also result from a change in the volume available for dilution. The turnover rate (TOR), or production rate of human CSF, exhibits physiological changes, for instance in relation to time of day [17]. It also decreases with aging in several species, including human [18,19], as well as in patients suffering from Alzheimer-type dementia [20]. We hypothesized that a similar mechanism could be involved in the physiological changes in the concentration of CSF steroids. To test this hypothesis, we assessed the kinetics of change in intra-ventricular pressure (IVP) after removal of a moderate volume of CSF, using a modified version of Masserman's method [21]. We estimated the TOR of the CSF three times after removing CSF in the same group of sheep, successively during periods of decreasing daylight, during constant long days and constant short days.

## Methods

The experiments were conducted in accordance with Authorization N°37801 for Animal Experimentation and Surgery from the French Ministry of Agriculture, following the European Community Council Directive 86/609/EEC. Surgery and postoperative care were conducted in certified facilities (Iso9001/2000 version, July 2006).

### Animals

*S*ix adult crossbred Romanov X Ile-de-France ewes aged 7 ± 1 yr were ovariectomized and received a 2 cm subcutaneous estradiol implant [22]. These implants induce a constant level of about 2 pg/ml of estradiol in the blood plasma, preserving the estradiol receptors in the brain but preventing physiological variations due to short-day estrous cycles. The ovariectomized, estradiol-treated ewes had been fitted three months earlier with a permanent intracerebral cannula in the third ventricle.

### Cannula implantation

Food and water was withheld for 24 h prior to premedication with atropine sulphate (20 mg, Chaix et Du Marais, Paris France). General anesthesia was induced by intravenous injection of barbiturate (Pentothal^R^; 12 μg/kg b.w., Abbott, Rungis, France) in order to perform tracheal intubation and was then maintained with isoflurane (3% in O_2_). Following the previously described method [23,15], the head was placed in a stereotaxic frame and after craniotomy, 1 ml of radio-opaque material (Omnipaque^R^, Nycomed Ingenon SA, Paris, France) was injected into the lateral ventricle through a cannula, delimiting the ventricular system on lateral and dorsal X-ray images. These lateral and dorsal images allowed us to place the cannula accurately within the third ventricle. The cannula (outer diameter 1.5 mm; inner diameter 0.8 mm) was constructed from a stainless steel luer-lock needle (Thibaud Biomedical Instruments, Thonon-Les Bains, France) and cut to a length of 40 mm. It was inserted into the third ventricle and the outflow of CSF through the cannula once inserted was checked. The cannula was then sealed and covered with a plastic cylinder. After surgery, the animals were allowed to recover for 4 days in a specifically designed room (heated, cushioned walls, and adapted to an artificial long-day or short-day regimen). During the recovery period, they received antibiotics (750 mg of amoxicillin; Clamoxyl^R^, Centravet Plancöet, France) and combined anti-inflammatory and diuretic treatment (250 mg of hydrochlorothiazide plus 2.5 mg of dexamethazone; Diurizone^R^, Centravet) daily.

### Intra-ventricular pressure (IVP) recording

IVP recording was performed between 2 and 4 h after lights-on in each light regimen. On the day before recording, the ewes were subjected to the same protocol of no food or water and with premedication and anesthesia as described above. As the sheep were in a 'Sphinx' position, i.e. with the head above the rest of the body, the cannula was connected to a pressure transducer (HARVARD Apparatus, Edenbridge, UK) initially set to zero. IVP measurements, averaging high and low values due to heart beat, were recorded every minute. After stabilization, a volume of 0.5 to 2 ml of CSF was removed with a 2 ml syringe via a three-way tap by gentle aspiration limited to 30 s and this volume was weighed to ensure precise measurement. Care was taken to ensure that the ventricle did not collapse; this was done by feel and as soon as any resistance to flow was felt we terminated the aspiration. The IVP (in mmHg) was then recorded for 15 min or more. Occasionally, the transducer was connected to a paper recorder and the IVP recorded in real time (not shown). After that, the sheep were allowed to recover from anesthesia and returned to the indoor, light-controlled pen.

### Experimental design

The IVP of the ewes was recorded first during the decreasing natural photoperiod at the September equinox (12 hL/12 hD), a time of year when gonadotropin secretion is stimulated (SG1). Thereafter, they were kept in artificial long days for a sufficient duration to ensure inhibition of gonadotropin secretion (IG) through increased negative feedback of estradiol at the level of the hypothalamus [13]. After 98 d of long-day treatment (16 hL/8 hD), they underwent the same protocol (anesthesia, CSF removal and IVP measurement). In one ewe, CSF could not be collected with the cannula during this long-day session, and it was therefore discarded from the analysis. The remaining ewes (n = 5) were then transferred to an artificial short-day photoperiod condition (8 hL/16 hD) for 82 d to recreate the same reproductive status as in SG1 and underwent the third protocol (SG2).

### Mathematical treatment

High and low IVP was measured continuously and averaged every minute. From the lowest IVP recorded, we measured the time taken to reach the initial pressure (T), and computed the average speed of recovery after CSF removal (slope in mmHg/min). The measurements were usually ended after 15 min. However, in two cases during the IG session, and one during SG2, IVP did not return to the initial values within this period. In these cases, the recording continued up to recovery (18 to 20 min) and a value of 15 min was attributed to the animals for computation. We computed the TOR of the CSF in μl/min by dividing the extracted volume of CSF by T. Analysis of differences between groups for the time changes of IVP after CSF removal was carried out using ANOVA, with effect of photoperiodic treatment and time as independent variables. Analysis of differences between groups for TOR was carried out using the Mann-Whitney non-parametric test. Values are given as mean ± SEM.

## Results

**Figure 1:**
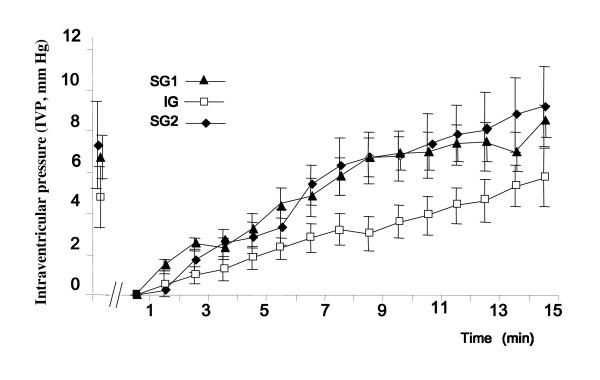
**Plots of IVP recorded before and after removal of CSF in adult sheep, from the lowest pressure recorded arbitrarily set to zero for homogeneity (means ± SEM, n = 5)**. Recordings were made during short-day stimulated reproductive status (SG1 and SG2), and long-day inhibited reproductive status (IG). Note the lower slope for recovery during IG than during SG1 and SG2 (interaction between time and IVP, ANOVA: *p *< 0.005, SG1 different from IG, *p *< 0.05).

**Figure 2:**
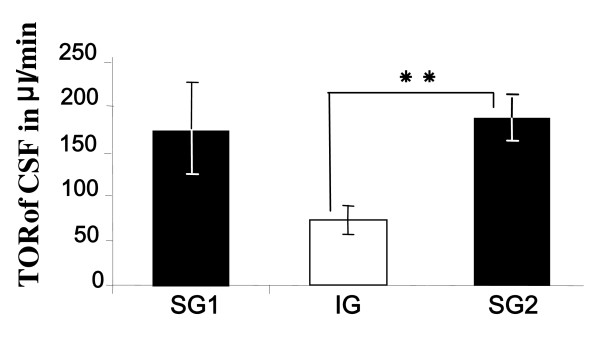
**Histograms of turnover rate (TOR) of CSF (Extracted volume in μl divided by the time for recovery of initial value in min) in the group of ewes during short days (SG1 and SG2) and long days (IG)**. * *: Mann-Whitney test, *p *= 0.016.

When the cannula was opened to connect the pressure transducer, slight CSF leaking (<0.2 ml) was occasionally, but not systematically observed, indicating IVP values very close to or equal to atmospheric pressure when the head of the animal was in the sphinx position. After removing the CSF, IVP in all animals first decreased within seconds and thereafter started to increase rapidly. When it reached the initial value, the IVP did not stabilize but went on increasing up to about 2 mmHg during the recording session. The kinetics for recovery of IVP differed between groups (Figure 1). The extracted volumes of CSF, i.e. the maximum that could be aspirated at those times, were 1.28 ± 0.36, 1.02 ± 0.26, and 1.73 ± 0.13 ml, and induced a fall in IVP of 5.33 ± 0.91, 4.16 ± 1.10, and 6.6 ± 1.72 mmHg, for SG1, IG and SG2, respectively (not significant). It is interesting to note that the fall in IVP was not individually correlated with the amount of CSF removed (correlation coefficient r^2 ^= 0.348), but was correlated with the speed of recovery (r^2 ^= 0.8171). The times (T) taken to reach the initial pressure were 8 min 30 sec ± 1 min 23 sec, 11 min 1 sec ± 54 sec and 9 min 0 sec ± 1 min 37 sec, for SG1, IG and SG2, respectively. IVP recoveries were quasi-linear from 2 to 15 min, with rates of recovery of 0.63 ± 0.07, 0.38 ± 0.06 and 0.72 ± 0.15 mmHg/min for SG1, IG and SG2 respectively. ANOVA showed a significant interaction for the time changes of IVP after CSF removal between treatment groups (SG1, IG and SG2, *p *< 0.005), with a statistically significant difference between SG1 and IG, *p *< 0.05. As shown in figure 2, the resulting TOR in SG2, 183.16 ± 23.82 μl/min, was not different from TOR in SG1, 169. 23 ± 51.58 μl/min (Mann-Whitney test, *p *= 0.41), but differed significantly from TOR in IG, 71.33 ± 16.59 μl/min (*p *= 0.016).

## Discussion

Our results demonstrate for the first time that IVP recovery after CSF removal varies with the photoperiodic status of the ewe. These results are in accordance with established data from other species, including human, suggesting a change in TOR in non-pathological situations, namely aging [18,19,24,25]. Interestingly, the value of TOR during SG2 measured after IG was not significantly different from TOR measured before IG, making it possible to eliminate a possible time-related loss in the reactivity of our experimental model. It is possible that the larger variation in TOR in SG1 than for SG2 could be due to the difference in light-dark cycles between the natural photoperiod which was decreasing in the month of September and the well controlled 98 long days of SG2. As TOR is calculated from time of recovery for IVP and the extracted volume of CSF, our data suggest an effect on the volume of CSF available at the time of sampling. Together, our results show that a higher TOR during short rather than long days could contribute to the lower concentration of CSF steroids observed in this situation [14,15] through a dilution mechanism. It therefore appears that not only the seasonal changes in hormone receptors shown previously [26,27], but also a change in concentration of their ligands could participate in the photoperiodic variations of the negative feedback of steroids on the hypothalamus. The comparable photoperiodic difference found in the concentration of the protein leptin in the CSF of male sheep could also involve this mechanism [16].

TOR values in sheep (112 μl/min: [28], 82 μl/min: [29]) have previously been published, but without taking into account the reproductive or photoperiodic status of the animals. They were obtained by a method of dilution based on ventriculocisternal perfusion, a method which has also been used with goats giving a comparable result (164 μl/min [30]). Although this method is accurate, it requires implantation of two cannulae and perfusion of exogenous molecules, which could have disrupted our protocol requiring long-term maintenance of the animals. However, it is striking that the TOR values obtained previously in sheep, and also in goats which have a similar brain size, are exactly within the range of those obtained during our experiment. Thus, measurement of IVP after CSF removal appears to be a valuable method of estimating and comparing TOR in sheep. The fall in IVP after CSF removal was correlated with speed of recovery rather than with the amount of CSF removed. This could indicate a seasonal effect on the compliance of the ventricular system, as suggested for age-related changes [18].

The previous studies were performed with un-anesthetized sheep or goats [28-30]. We are aware that anesthesia could have interfered with the measurements in our experiment. First, as the time after cannula implantation and between IVP measurements ranged from two to three months, it is unlikely that the post-operative drugs affected CSF secretion. With regard to the use of anesthesia during the measurements, while the literature shows the unavoidable impact of anesthesia on CSF secretion and elimination, there is no evidence to our knowledge of any interaction between anaesthesia and photoperiodic effects. In the study by Silverberg [20], patients with Alzheimer's disease showed a 50% decrease in the TOR of CSF when compared to sedated patients with Parkinson's disease. In that study, the Alzheimer's patients had been anesthetized using various drugs, some supplemented with 0.5% isoflurane, but the authors stated that this treatment did not appear to affect the results significantly. Isoflurane does not affect CSF production, but dosage can change the reabsorption differentially [31]. Very recently, it has also been shown that isoflurane at 3% could open the human blood-brain barrier [32], potentially changing the CSF. In our animal model, IVP recordings were taken from three un-anesthetized sheep under long-day conditions, demonstrating some comparable kinetics of recovery, but movement artifacts and possible stress meant that this method was not reliable. Regardless of the possible effect of anesthesia, our measurements were comparable between reproducible situations in our experiment.

Daily changes in the TOR of human CSF have been described, with increased TOR at night. This nocturnal increase in CSF production is inhibited by a β1-receptor antagonist, a type of receptor stimulating melatonin secretion in the pineal gland [33]. Our experiments, performed two to four hours after lights-on during short and long days did not allow this putative daily regulation to be checked in sheep. Nonetheless, photoperiodic as well as daily regulations are mostly linked to melatonin secretion from the pineal gland [34]. In our previous study, we showed that the photoperiodic differential concentration of estradiol in the CSF was pineal-dependent [15]. High melatonin concentrations [35], conveying photoperiodic information directly from the pineal gland to the ventricular system [9] and binding to the choroid plexus, have been found in the CSF of several species, including ungulates [36-38]. This neuro-hormone also stimulates the secretory activity of the choroid plexus in the hamster and rat [39,40]. Interestingly, while our studies indicate changes in permeability and TOR of CSF in photoperiodic animals, others identify the choroid plexus as an 'active filter' for penetration of specific liver-borne proteins in the brain of chipmunks during hibernation, another melatonin-related function [41]. Thus, we could postulate that melatonin modulate the functions of the choroid plexus [42]. A previous study using Madin-Darby canine kidney (MDCK) cells as a model of cultured polarized epithelial cells with tight junctions resembling the epithelial cells of the choroid plexus showed that melatonin induces cyclic, circadian variations of water transport [43]. This study postulates an effect of melatonin on the tight junctions through PKC phosphorylation of actin, a mechanism which could perhaps be similarly involved in changes in CSF production by the choroid plexus.

## Conclusion

Using a light-dark cycle which reproduces the photoperiodic modulation of reproductive activity, this study has shown that the turnover rate of the CSF in the ewe is higher during periods of short day length and reduced in periods of long day length. This phenomenon could account for the higher concentration of estradiol in the CSF, previously observed during long days when the estrous cycle is blocked by increased negative feedback of this steroid in the hypothalamus. Our study highlights the active role of the choroid plexus and CSF in neuroendocrine regulation.

## List of abbreviations used

IG: inhibitory period of gonadotropin activity; IVP: intra-ventricular pressure; PKC: Protein kinase C; SG1 and SG2: first and second stimulatory periods of gonadotropin activity; TOR: CSF turnover rate.

## Competing interests

The authors declare that they have no competing interests.

## Authors' contributions

JCT conceived and designed the project and drafted the manuscript. JCT, DL and SB carried out the experiment and collected data. BM was involved with interpretation and mathematical processing of data and drafted the manuscript. JCT and BM revised the manuscript. All the authors read and approved the manuscript.
